# Standardization of Diagnostic Biomarker Concentrations in Urine: The Hematuria Caveat

**DOI:** 10.1371/journal.pone.0053354

**Published:** 2012-12-31

**Authors:** Cherith N. Reid, Michael Stevenson, Funso Abogunrin, Mark W. Ruddock, Frank Emmert-Streib, John V. Lamont, Kate E. Williamson

**Affiliations:** 1 Molecular Biology, Randox Laboratories, Crumlin, Northern Ireland; 2 Department of Epidemiology and Public Health, Queens University Belfast; Belfast, Northern Ireland; 3 Centre for Cancer Research & Cell Biology, Queens University Belfast, Belfast, Northern Ireland; The University of Texas M. D. Anderson Cancer Center, United States of America

## Abstract

Sensitive and specific urinary biomarkers can improve patient outcomes in many diseases through informing early diagnosis. Unfortunately, to date, the accuracy and translation of diagnostic urinary biomarkers into clinical practice has been disappointing. We believe this may be due to inappropriate standardization of diagnostic urinary biomarkers. Our objective was therefore to characterize the effects of standardizing urinary levels of IL-6, IL-8, and VEGF using the commonly applied standards namely urinary creatinine, osmolarity and protein. First, we report results based on the biomarker levels measured in 120 hematuric patients, 80 with pathologically confirmed bladder cancer, 27 with confounding pathologies and 13 in whom no underlying cause for their hematuria was identified, designated “no diagnosis”. Protein levels were related to final diagnostic categories (p = 0.022, ANOVA). Osmolarity (mean = 529 mOsm; median = 528 mOsm) was normally distributed, while creatinine (mean = 10163 µmol/l, median = 9350 µmol/l) and protein (0.3297, 0.1155 mg/ml) distributions were not. When we compared AUROCs for IL-6, IL-8 and VEGF levels, we found that protein standardized levels consistently resulted in the lowest AUROCs. The latter suggests that protein standardization attenuates the “true” differences in biomarker levels across controls and bladder cancer samples. Second, in 72 hematuric patients; 48 bladder cancer and 24 controls, in whom urine samples had been collected on recruitment and at follow-up (median = 11 (1 to 20 months)), we demonstrate that protein levels were approximately 24% lower at follow-up (Bland Altman plots). There was an association between differences in individual biomarkers and differences in protein levels over time, particularly in control patients. Collectively, our findings identify caveats intrinsic to the common practice of protein standardization in biomarker discovery studies conducted on urine, particularly in patients with hematuria.

## Introduction

Advances in proteomics have enhanced our understanding of the urinary proteome [1]–[4] and subsequently encouraged biomarker discovery screens in a range of complex diseases [2], [3], including bladder cancer [5], [6]. Urine has the advantage of ease of access and is relatively stable thermodynamically [3]. Despite these encouraging developments, no biomarker or biomarker combination to date, has achieved widespread clinical application as a diagnostic assay. Perhaps this is partly attributable to the range of methodologies used to standardise urinary biomarker levels which introduces a lack of consistency in reported levels and inhibits cross study comparisons.

When we reviewed publications on biomarkers for urological pathologies to ascertain the ‘correct’ methodology to employ for urine normalization, we found inconsistency. As there is no standard methodology, the normalization method employed for any given study is still very much at the discretion of the project investigator, the accessibility of equipment and the available technical expertise. Further, insufficient research into the effects of different standardization approaches means that researchers are employing methods which may introduce bias. Thus there is the potential both for biased data and masking detection of valuable biomarkers secreted into urine at low levels [7].

Some researchers have reported biomarker levels in units per unit volume of urine [5], [8], [9]; others have standardized biomarker levels using urinary creatinine [10]–[13]. Most, however, have opted to use protein as their denominator [5], [14]–[16]. Creatinine, the breakdown product of creatine phosphate during muscle metabolism, is filtered out of the blood into the urine by the kidney. Creatinine production is usually at a fairly constant rate when renal function, metabolism and muscle mass are stable, but can be dependent on age, sex, race and size [17]. Serum creatinine and the albumin:creatinine ratio in urine are in clinical use as biomarkers of kidney disease [18]. Osmolarity is a measure of the osmoles of solute per litre of solution and therefore reflects the concentrating ability of the kidneys. Protein is often used to normalize potential bladder cancer biomarkers [5], [14]–[16]. Proteinuria is, however, synonymous with diabetes and renal diseases [7], [18]–[20].

Potential biomarkers must proceed through rigorous validation before they progress through the phases that span discovery to clinical application [21]. However, in the absence of evidence-based guidelines for the standardization of urinary biomarkers, it is possible that potential biomarkers secreted at low levels into urine have not been identified. Urine standardization guidelines would complement those already established for Standards for Reporting of Diagnostic Accuracy (STARD) [22], [23] and would ensure that promising biomarkers could be cross-referenced thus facilitating their more expeditious development. It is, however, conceivable that individual guidelines tailored to the specifics of different confounding factors may be required.

The aim of this study was to increase our understanding about the consequences and effects of different methods employed to standardize biomarker levels detected in urine collected from hematuric patients. We assessed the effects on three biomarkers previously reported to be associated with bladder cancer i.e., interleukin- 6 (IL-6) [24], IL-8 [25] and vascular endothelial growth factor (VEGF) [26]. Using data collected during a case control study [27], we characterized urinary creatinine, osmolarity and protein levels across patient groups with the following final diagnoses: no diagnosis (n = 13), confounding pathologies (n = 27) and bladder cancer (n = 80). We determined areas under the receiver operator characteristic (AUROC) for IL-6, IL-8 and VEGF both for uncorrected data and for data standardized using urinary creatinine, osmolarity or protein. In 72 hematuric patients, we compared the intra-patient variability of levels measured at recruitment and at follow-up. We assessed whether there was any association between the differences in levels of biomarkers on recruitment and those measured at follow-up and the differences similarly detected in levels of the standards in the same samples. We present findings that indicate urine volume standardization is preferable to the use of protein standardization because of the high incidence of proteinuria in the hematuric patient population.

## Methods

### Patient Samples

A case control study approved by the Research Ethics Committee, Faculty of Medicine, Queen’s University Belfast (80/04) and the Office for Research Ethics Committees Northern Ireland (ORECNI 80/04); and reviewed by the Belfast City Hospital Trust review board and the Ulster Community and Hospital Trust Research Committee was conducted according to STARD guidelines [22], [23]. Written consent was obtained from 181 patients with hematuria (103 patients with confirmed transitional cell carcinoma; and 78 controls); recruited between November 2006 and October 2008 [27]. All patients were white Caucasians except for one of black African origin. Dipstick analysis is a simple and fast analyses of urine undertaken by medical personnel to determine the levels of constituents in urine, including blood, protein, and white blood cells. Dipstick analyses were undertaken on urine samples collected from each of the patients using Aution Sticks 10EA, which were interpreted using PocketChem (Arkray factory, Inc. Japan). The dipstick results for protein were recorded. Approximately 250 mg/l (0.25 mg/ml) is the lower limit of sensitivity for urine dipstick testing [20]. Urine samples from each patient were then stored at –80°C for a maximum of 12 months prior to triplicate analyses of urinary creatinine, osmolarity, protein, IL-6, IL-8 and VEGF.

First, we analysed data from 120/181 hematuric patients (96 males:24 females) with a mean age = 66 years. Eighty of these patients had pathologically confirmed transitional cell carcinoma of the bladder (TCCB) and 40 were controls. Of the controls, 27 had confounding pathologies, such as stones, inflammation or benign prostate enlargement. In 13 patients, even after detailed investigations, including cystoscopy and radiological imaging of the upper urinary tract, no underlying cause for their hematuria was identified. The diagnosis for these patients is referred to as “no diagnosis”.

In our second set of analyses, we compared standards and biomarker levels across time in 72/181 patients (60 males: 12 females) with a mean age = 69 years. Urine samples were collected from these 72 patients both at the time of recruitment and at a second visit (median interval = 11 months (range 1 to 20 months)). It was not possible to collect longitudinal samples from all 181 patients recruited to the study because many patients had significant distances to travel to hospital. The characteristics of these 72 patients were representative of the 120 patients previously analysed. Forty-eight of these patients had TCCB. Sixteen of the 24 controls had confounding pathologies and 8 had a final diagnosis of no diagnosis.

### Creatinine, Osmolarity, Protein, IL-6, IL-8 and VEGF Analyses

Scientists, blinded to patient data, completed triplicate analyses of urine samples at Randox Laboratories Ltd. Creatinine levels (µmol/L) and osmolarity (mOsm) were measured using a Daytona RX Series Clinical Analyser (Randox) and a Löser Micro-Osmometer (Type 15) (Löser Messtechnik, Germany), respectively [28], [29]. Total protein levels (mg/ml) in urine were determined by Bradford assay A_595 nm_ (Hitachi U2800 spectrophotometer) using Bovine Serum Albumin (BSA) as standard. IL-6, IL-8 and VEGF (pg/ml) levels in urine (sensitivity = 1.6, 7.9 and 14.6 pg/ml, respectively) were measured [30] using Randox Biochip Array Technology (Randox Evidence © and Investigator ©), which are multiplex systems for protein analysis [31].

### SDS PAGE Analyses

Urine samples (2.5 µl/lane) from each patient were investigated for protein using SDS PAGE (16%) analysis. The gels were stained with Coomassie Blue for 1 h and then de-stained in methanol/acetic acid/water (2∶1∶7) until clear. Protein bands, observed for each patient, were quantified using Quantiscan © software.

### Statistical Analyses

Using data from the 120 hematuric patients, we assessed the distribution of the three standards by visual comparison of histograms and boxplots and interpretation of means, medians, skewness and kurtosis. We explored correlations and then used linear regression to determine the extent to which one standard could predict another. To determine the relationship between protein levels measured in urine and protein categories defined using dipstick analyses, we examined a scatter plot of protein levels (mg/ml) against dipstick protein categories to ascertain the range of protein levels within each dipstick category i.e. “+”, “++”, “+++” and “++++”. We used one-way ANOVA to determine whether protein levels were related to final diagnoses categories. To ascertain their diagnostic potential, we compared the area under the receiver operating characteristic (AUROC) determined for the standardized and uncorrected biomarker levels. We divided the average measurement for each of the three biomarkers by the average osmolarity, creatinine or protein level measured in the same patient’s urine sample and then log_10_ transformed the data.

Urine samples were obtained from 72 patients on two visits; one on recruitment and a second at follow-up (median = 11 (1 to 20 months)). To assess the agreement between the levels of the standards on recruitment and at follow-up, we constructed Bland Altman plots and undertook paired t-test analyses. In addition, we were interested to ascertain whether there were significant associations between differences in individual biomarkers levels over time and differences in standard levels over time. For each biomarker we divided the mean biomarker level measured at follow-up by the mean biomarker level measured on recruitment, and then computed the log_10_ of this value. Similarly, for each standard we divided the mean level measured at follow-up by the mean level measured at recruitment and then computed the log_10_ of this value. To compare these ratios we undertook regression analyses inserting log differences of each biomarker into the dependent box and log differences of creatinine, osmolarity and protein sequentially into the independent box.

Statistical analyses were completed using SPSS v17.

## Results

**Figure 1:**
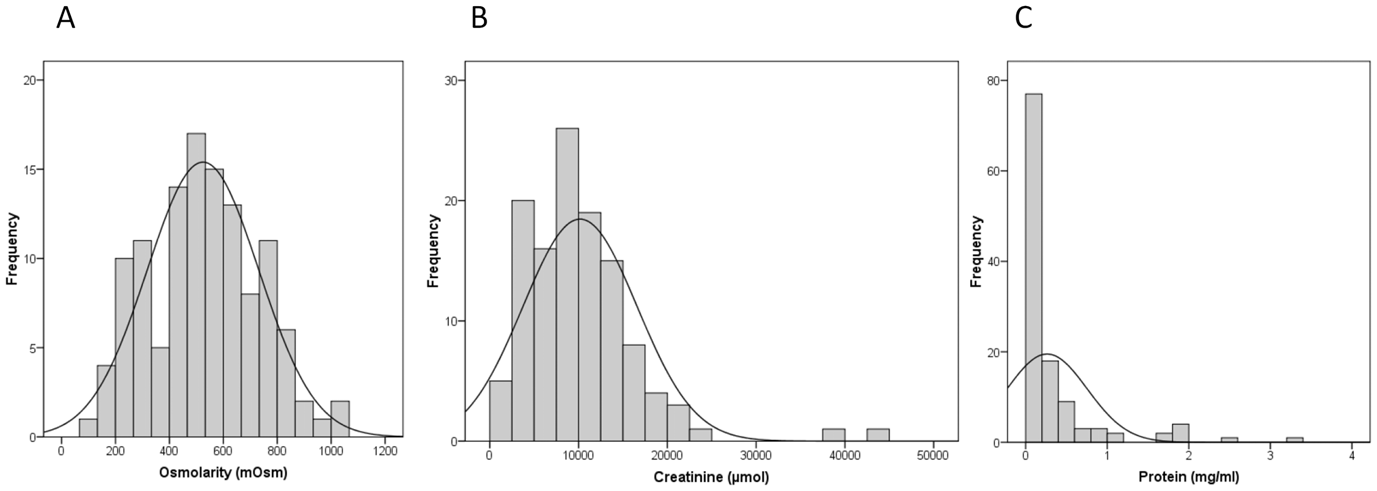
Creatinine, Osmolarity and Protein distributions. Triplicate levels of the standards were measured in 120 hematuric patients and then averaged. (A) Osmolarity was normally distributed; (B) creatinine and (C) protein had skewed distributions.

**Figure 2:**
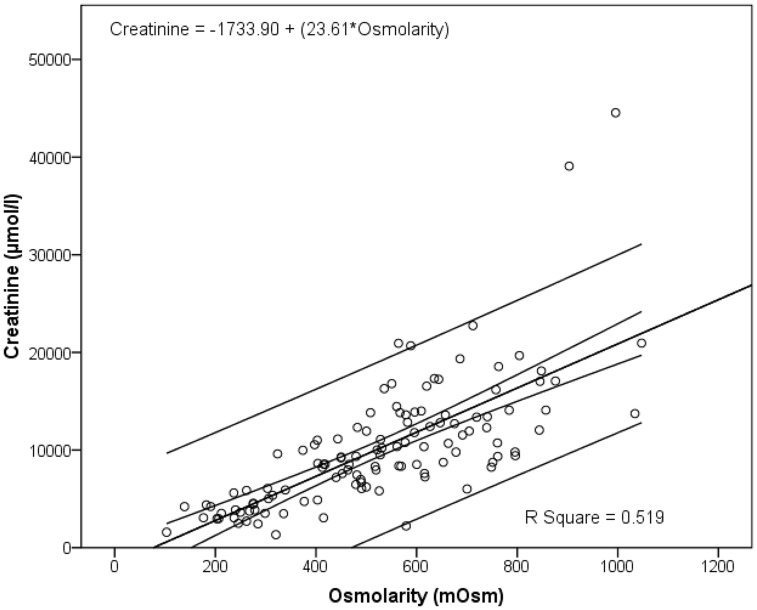
Relationship between osmolarity and creatinine. Triplicate levels of osmolarity and creatinine were measured in urine from 119 hematuric patients. There was a modest relationship between osmolarity and creatinine (R Square = 0.519).

**Figure 3:**
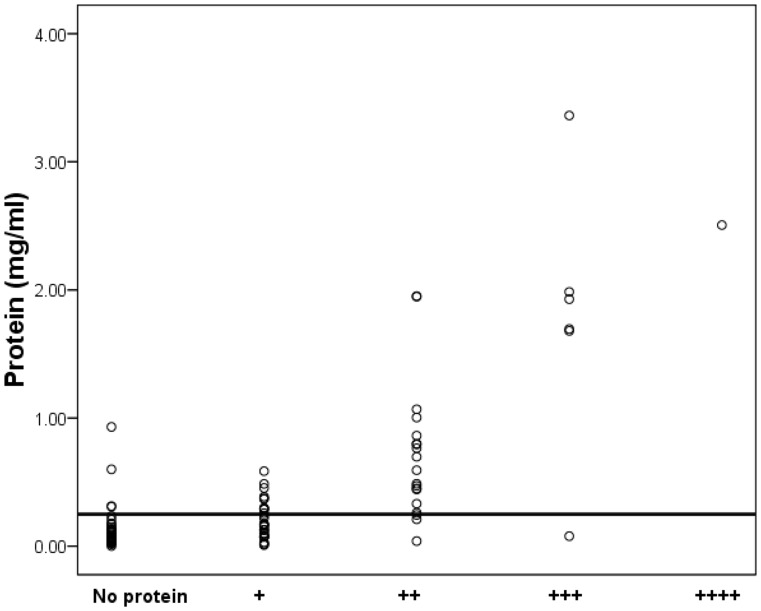
Comparison between measured protein levels and protein dipstick analyses. Total protein levels (mg/ml) in urine were determined by Bradford assay A_595 nm_ (Hitachi U2800 spectrophotometer) using Bovine Serum Albumin as standard. Dipstick analyses were undertaken using Aution Sticks 10EA. Analyses were interpreted using PocketChem (Arkray factory, Inc. Japan). Protein levels were plotted against dipstick results with the Y –axis reference line indicating the usual lower limit of sensitivity for urine dipstick testing (0.25 mg/ml).

Osmolarity (mean = 529 mOsm; median = 528 mOsm) was normally distributed while creatinine (mean = 10163 µmol/l, median = 9350 µmol/l) and protein (mean = 0.3297 mg/ml; median = 0.1155 mg/ml) distributions were not. This is substantiated by skewness and kurtosis values for osmolarity (0.1; −0.5), creatinine (2.2; 8.8), and protein (3.1; 10.6) (Figure 1). Measurements for osmolarity, creatinine, and protein ranged from 103 to 1047 mOsm; 1329 to 44542 µmol/l (1.3 to 44.5 mmol); and zero to 3.36 mg/ml, respectively. Two patients had extreme creatinine levels (Figure 1B). These levels, 44542 and 39077 µmol/l respectively, were measured in a 40 year-old male with stone disease and a 58 year-old male with non-muscle invasive TCCB. All other measurements were <24000 µmol/l. Extreme creatinine levels have been reported previously [12]. There was a modest relationship between osmolarity and creatinine in that 51.9% of the variation in creatinine was accounted for by osmolarity (linear regression; R Square = 0.519) (Figure 2). In this study we report 49% false positives and <1% false negatives in dipstick analyses based on our findings that 25/51 patients deemed dipstick positive had protein levels <0.25 mg/ml; and that 4/62 patients with measured protein levels >0.25 mg/ml were deemed dipstick negative (Figure 3).

Urinary protein levels were related to final diagnoses categories (ANOVA; p = 0.022). Protein levels in urine from bladder cancer patients were higher than in those with no diagnosis (p = 0.073)(Table 1). In contrast, osmolarity and creatinine levels were not significantly related to final diagnoses (ANOVA p = 0.851 and 0.630, respectively).

**Table 1:** Comparison of protein levels across final diagnostic categories.

	(I) finaldiagnosticcategory	(J) finaldiagnosticcategory	MeanDifference(I–J)	Std. Error	Sig.	95% CILowerbound	95% CIUpperbound
Dunnett T3	no diagnosis	confounding pathologies	−.33166	.20616	.316	−.8650	.2017
		bladder cancer	−.46508	.18809	.073	−.9667	.0366
	confounding pathologies	no diagnosis	.33166	.20616	.316	−.2017	.8650
		bladder cancer	−.13342	.12194	.621	−.4352	.1684
	bladder cancer	no diagnosis	.46508	.18809	.073	−.0366	.9667
		Confounding pathologies	.13342	.12194	.621	−.1684	.4352

Urinary protein levels measured in 120 patients with hematuria were related to final diagnostic categories in (ANOVA; p = 0.022). Subsequently, we carried out a one way ANOVA with post-hoc Dunnett T3 analyses using log_10_ transformed protein data. Higher protein levels were measured in urine from patients diagnosed with bladder cancer in comparison to those with no diagnosis (p = 0.073). There were no significant differences between the protein levels measured in patients with confounding pathologies and levels measured in the urines from bladder cancer patients (p = 0.621) or between patients with no diagnosis and patients with confounding pathologies (p = 0.316).

**Figure 4:**
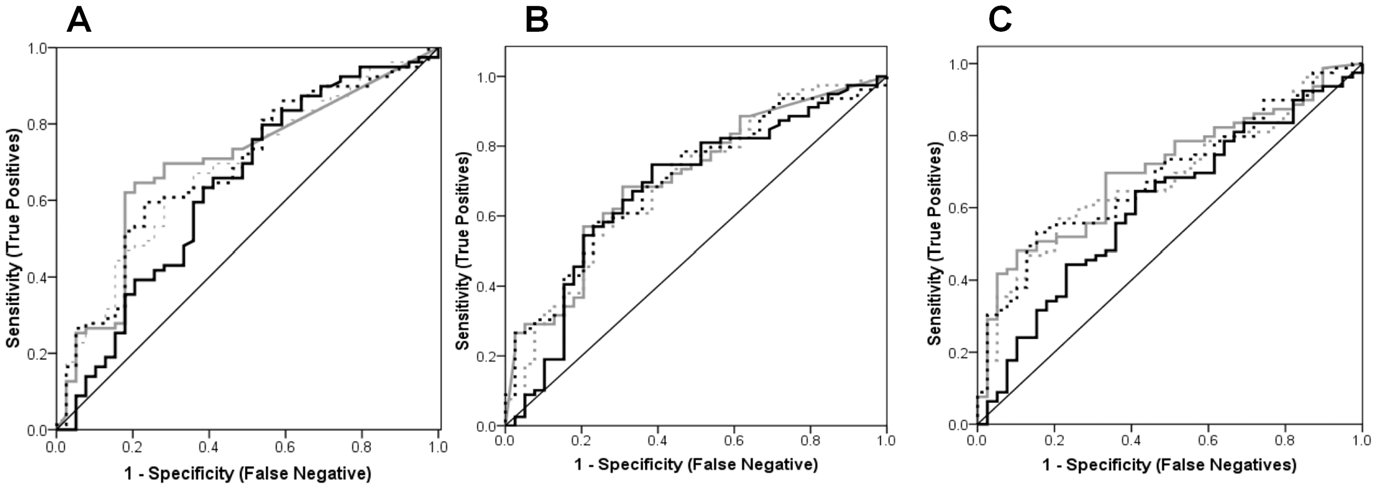
AUROC for IL-6, IL-8 and VEGF. The lowest area under receiver operating characteristic (AUROC) were determined after protein normalization as represented by the solid black curve which was always closest to the diagonal reference line i.e., IL-6 = 0.634 (0.523 to 0.745); IL-8 = 0.677 (0.570 to 0.784); and VEGF = 0.609 (0.501 to 0.716). The AUROCs for uncorrected biomarker levels (thick grey curve), and those standardized using osmolarity (dashed black curve) or creatinine (dashed grey curve) were very similar for individual biomarkers : (A) IL-6 = 0.693 (0.592 to 0.794), 0.683 (0.582 to 0.784) and 0.678 (0.578 to 0.779), respectively; (B) IL-8 = 0.706 (0.608 to 0.804), 0.701 (0.603 to 0.799) and 0.694 (0.592 to 0.795), respectively; and (C) VEGF = 0.705 (0.610 to 0.799), 0.687 (0.591 to 0.783) and 0.680 (0.583 to 0.777), respectively. Figures in brackets are 95% Confidence Intervals.

The ranges and median levels for the biomarkers were: IL-6 (pg/ml) (n = 119)(range = 1.2 to 900.0; median = 3.0), IL-8 (pg/ml) (n = 119)(range 7.90 to 2900.0; median = 117.3) and VEGF (pg/ml)(n = 119)(range = 14.6 to 1500.0; median = 107.6). AUROCs for uncorrected biomarker levels and those standardized using osmolarity or creatinine were very similar. The lowest AUROCs were consistently recorded following protein standardization (Figure 4).

**Figure 5:**
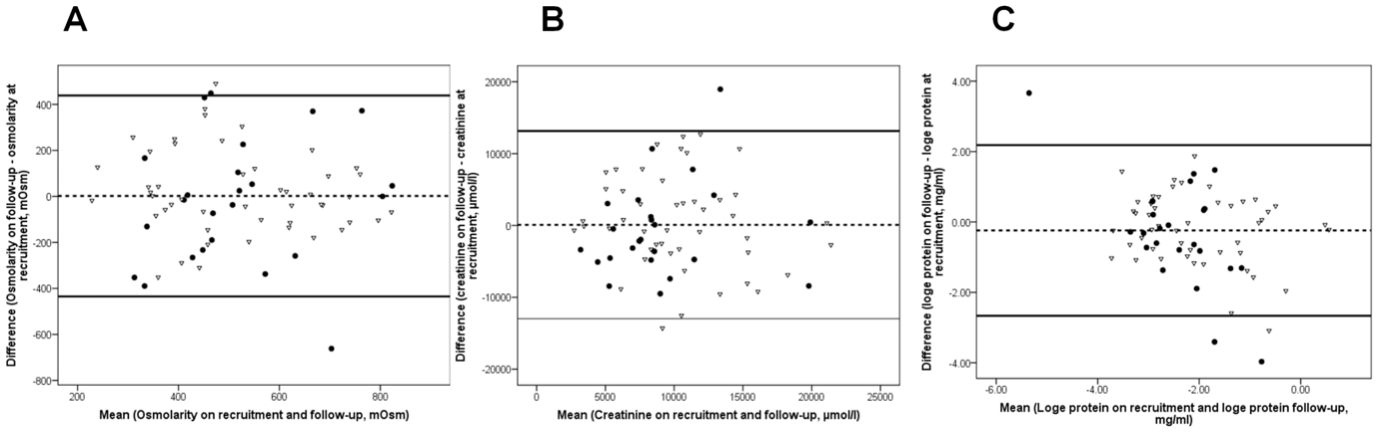
Bland Altman plots for osmolarity, creatinine and protein. Bland Altman plots for (A) osmolarity; (B) creatinine; and (C) protein (log_e_) were plotted to determine the agreement between the levels of each standard measured on recruitment and those measured at follow-up. The hashed line (mean of the mean differences) demonstrates that protein levels decreased by approximately 24% at follow-up. Osmolarity and creatinine levels did not significantly change. Solid lines, 95% CI limits. Open triangles (bladder cancer); closed black circles (controls).

Median protein levels were lower at follow-up (0.08 mg/ml) when compared to levels on recruitment levels (0.10 mg/ml). Osmolarity and creatinine were constant. Mean osmolarity = 519, 521 mOsm; mean creatinine = 9835, 9941 µmol/l, respectively on recruitment and at follow-up. Median osmolarity = 527, 515 mOsm; median creatinine = 9086, 8832 µmol/l, respectively on recruitment and at follow-up. Bland Altman plots illustrated that protein levels decreased by approximately 24% between recruitment and follow-up (mean log_e_difference = −0.24 (95% Confidence Interval (CI) 2.18 to −2.66). In contrast, osmolarity and creatinine were stable with little variation across the scale in the Bland Altman plots (Figure 5). Protein levels decreased between recruitment and follow-up (Paired T-test; p<0.10) (Table 2).

**Table 2:** Paired t-test comparing standard levels measured on recruitment and at follow-up.

		Mean	Std.Deviation	Std.Error Mean	95% CILower	95% CIUpper	t	df	Sig.(2-tailed)
Pair 1	log_10_ protein on recruitment – log_10_ protein at follow-up	.10451	.52658	.06206	−.01923	.22825	1.684	71	.097
Pair 2	creatinine on recruitment – creatinine at follow-up	−87.45193	6531.78735	775.18054	−1633.50077	1458.59692	−.113	70	.911
Pair 3	osmol arity on recruitment- osmolarity at follow-up	−1.75926	218.40565	25.73935	−53.08207	49.56355	−.068	71	.946

Urine samples were obtained on two visits; one on recruitment and a second at follow-up (median = 11 (1 to 20 months)) from 72 patients who had presented with hematuria. The mean difference between log10 protein levels decreased over time (p = 0.097).

**Figure 6:**
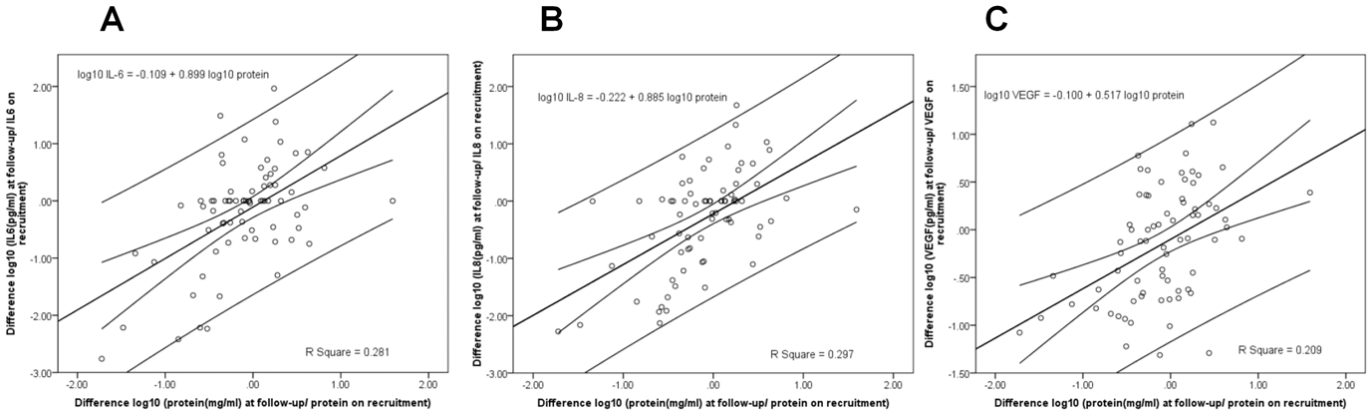
Regression analyses to determine the relationship between differences in standards and biomarkers over time. Scatter plots, based on data from 72 hematuric patients, plotting the differences between biomarker levels on recruitment and follow-up against the differences between protein levels on recruitment and follow-up for (A) IL-6, (B) IL-8 and (C) VEGF. The regression line and 95% confidence interval show significant associations (p<0.0001 for all biomarkers). Differences in biomarker levels across time were associated with differences in protein levels.

When we studied longitudinal ratios there were significant associations between the differences in logarithms (base 10) between all three biomarkers and protein. These associations between the biomarkers and protein ratios were stronger in the control sub-population (n = 24) than in the bladder cancer sub-population (n = 48). In the control sub-population, IL-6 = −0.55+0.739 protein, R Square = 0.318 (p = 0.004); IL-8 = −0.231+0.848 protein, R Square = 0.318 (p = 0.004); and VEGF = −0.075+0.477 protein, R Square = 0.322 (p = 0.004). In the bladder cancer sub-population, IL-6 = −0.130+1.099 protein, R Square = 0.285 (p = <0.0001); IL-8 = −0.216+0.928 protein, R Square = 0.278 (p = <0.0001); and VEGF = −0.111+0.569 protein, R Square = 0.165 (p = 0.0001). There were no significant associations when recruitment levels were subtracted from follow-up levels of biomarkers and the differences similarly determined in either osmolarity or creatinine in the same samples (Figure 6).

**Figure 7:**
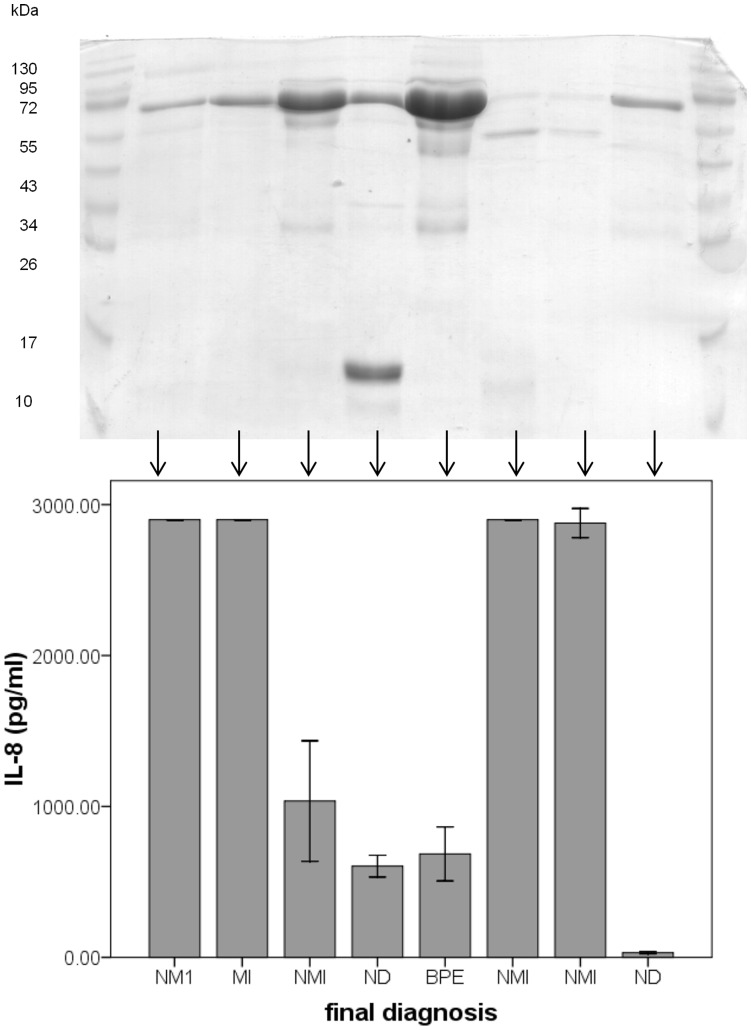
SDS PAGE on urine samples. SDS PAGE was carried out on urine from each patient. A dense band was frequently observed at approximately 64–66 kDa. This band represents albumin. Eight representative samples demonstrate the diverse relationship between this albumin band on the SDS PAGE and corresponding IL-8 levels measured in urine from the same patient sample. Corresponding IL-8 levels are illustrated in the 95% confidence limit error bar chart directly below each lane. The density of the albumin band was not always indicative of the IL-8 levels. Four patients had non-muscle invasive bladder cancer (NMI), one patient had muscle invasive bladder cancer (MI), two patients had no diagnosis (ND), and one patient had benign prostate enlargement.

We analysed the urine from each patient using PAGE. The levels of protein in the urine that we observed on the gel, following equal loading (2.5 µl, i.e. no standardization or normalization), did not reflect the level of the biomarker in the same urine sample. Therefore high levels of protein observed on the PAGE gel did not correlate with high levels of the biomarkers. For example, IL-8 levels did not significantly correlate with the band density frequently observed at approximately 64–66 kDa (Figure 7).

## Discussion

We have presented evidence that the high prevalence of proteinuria in hematuric patients introduces a caveat with respect to using protein as the standardiser of urinary biomarker levels. The origin of proteins shed into the urine of patients with proteinuria is dependent on the specific disorder that the patient has [7]. Further, drugs which are often prescribed for hematuric patients, including nonsteroidal anti-inflammatory and occasionally angiotensin-converting enzyme (ACE) drugs, can cause increases or decreases in proteinuria [32]. In certain renal diseases large proteins such as albumin leak into urinary space and the amount of secreted protein very much depends on the specific disease [7]. Dipstick protein analyses detects predominantly albumin. Proteinuria is classified as selective when albumin is the major protein constituent [7]. Albumin is detected as a dense band at approximately 64 to 66 kDa observed on the SDS PAGE gel indicating that the corresponding patients have selective proteinuria. In contrast, the patient with a dense band around 13 kDa may have non-selective proteinuria. There was a significant correlation r = 0.802 (Pearson correlation; p<001) between the density of the albumin band quantified using Quantiscan © software and log_10_ average protein levels, but this, on its own, would not justify the classification of patients with proteinuria as having albuminuria.

This study has therefore demonstrated in three ways the caveats of protein normalization in patients with hematuria. These being that protein levels are not homogeneous across diagnostic groupings in hematuric patients; that there is intra-patient variability in protein levels in urine over time; and that protein standardization reduced AUROCs in biomarkers previously demonstrated to be elevated in bladder cancer patients [24]–[26]. First, we have demonstrated that urinary protein levels were higher in patients with bladder cancer compared to those with no final diagnosis and that protein *per se* is associated with final diagnosis. Second, we found that standardization using protein resulted in the lowest AUROCs for each of the three bladder cancer diagnostic biomarkers. The latter indicates that biomarker differences between controls and bladder cancer patients can be attenuated following protein standardization. Third, we observed that protein levels were generally lower on follow-up, perhaps indicative of successful treatment. However, there were significant associations between the differences determined in each of in the biomarkers when recruitment levels were subtracted from follow-up levels and the differences similarly determined in protein in the same samples. This was most evident in controls. These findings suggest that after treatment and/or recovery, protein levels decreased in the control sub-population to a greater extent than in the cancer patients. This finding would only arise if controls were not healthy and controls in some case control studies would be healthy and therefore protein concentration would not be expected to be lower at the end of the study. The latter associations would support the use of protein normalization, particularly in controls. However, in light of other findings, particularly considering that the lowest AUROCs were determined following protein normalization and the high prevalence of proteinuria in this patient population, this approach could bias true biomarker levels.

These observations demonstrate that it is not appropriate to use protein standardization of urine samples in hematuric patient populations where proteinuria is often a co-morbidity. However, our findings cannot be extrapolated to patients who have proteinuria who are nonhematuric. Proteinuria has widespread causes including ureteric calculi, minimal change glomerulo-nephritis, diabetes, malaria and congestive heart failure [7], [33]. Many of these pathologies present with hematuria [27].

Interestingly, differences in protein levels between recruitment and follow-up accounted for a significant amount of the differences in biomarker levels at the same time-points. Further this relationship was strongest in the control samples. This reflects a close relationship between a disease status indicator, i.e. proteinuria, and IL-6, IL-8 and VEGF levels in Patients with hematuria.

The persistent trend for researchers to normalize biomarker levels using protein [5], [14]–[16] perhaps stems from the concept of equal loading in Western blot experiments, which has been carried through, to biomarker studies and then more recently, to proteomic screens. It is interesting that Chen *et al* (2010) achieved higher AUROCs for novel potential bladder cancer- biomarkers using urine volumes rather than protein normalized samples [5]. Our data suggest that in hematuric populations in which there is a high incidence of proteinuria, urine volume is likely to be more accurate, and indeed a simpler approach to standardization, than applying protein as a denominator. The consequences of normalizing using protein in this study were that biomarker levels in patients with proteinuria were proportionately reduced. This approach therefore introduced bias. It might be prudent to consider proteinuria as a contraindication to protein based standardization of urine in proteomic studies conducted in patients with hematuria. In other biomarker applications different confounding pathologies may play a role and our findings might not apply [7].

This is the first time that the effects of biomarker standardization have been compared across four methodologies simultaneously, i.e. uncorrected levels, creatinine, osmolarity and protein. Standardization of the urinary biomarker levels using protein, attenuated the data reducing both sensitivity and specificity of the biomarkers IL-6, IL-8 and VEGF. In this study, urinary creatinine and osmolarity levels in patients were constant in patients over time. Since creatinine and osmolarity did not differ significantly across disease pathologies frequently diagnosed in hematuric patients, our data suggest that creatinine or osmolarity could be used to normalize for urinary protein biomarkers. Further, osmolarity levels measured in this study predicted creatinine levels supporting the notion that osmolarity and creatinine levels in urine are interchangeable. However differences in IL-6, IL-8 and VEGF measured on recruitment and at follow-up were not significantly associated with differences in either of these standards. In this study we did not evaluate the efficacy of standardization based on 24 hour urine collections which might be more accurate than the state measurements used in this study. This study provides no justification for normalization using either creatinine or osmolarity when they are determined as state measurements. Uncorrected IL-6, IL-8 and VEGF AUROC analyses were very similar to those normalized using osmolarity and creatinine. Therefore, it makes more sense to use uncorrected biomarker levels for biomarker studies in hematuric patients.

Our study provides evidence that urinary diagnostic biomarkers should be standardized by urine volume in hematuric patients where there is a high incidence of proteinuria. Since proteinuria is a common condition in patients with hypertension, ureteric calculi, minimal change glomerulo-nephritis, diabetes, malaria and congestive heart failure, our findings may have implications for a wide range of biomarker discovery, biomarker validation and quantitative proteomic studies investigating complex diseases.
